# Robot-assisted radical cystectomy and intracorporeal neobladder formation: on the way to a standardized procedure

**DOI:** 10.1186/1477-7819-13-3

**Published:** 2015-01-06

**Authors:** Christian Schwentner, Allen Sim, Mevlana Derya Balbay, Tilman Todenhöfer, Stefan Aufderklamm, Omar Halalsheh, Johannes Mischinger, Johannes Böttge, Steffen Rausch, Simone Bier, Arnulf Stenzl, Georgios Gakis, Abdullah Erdem Canda

**Affiliations:** Department of Urology, Eberhard-Karls University, Hoppe-Seyler Str. 3, 72076 Tübingen, Germany; Department of Urology, Memorial Şişli Hospital, Istanbul, Turkey; Department of Urology, Ankara Atatürk Training & Research Hospital, Ankara, Turkey

**Keywords:** intracorporeal diversion, laparoscopy, neobladder, radical cystectomy, robot-assisted

## Abstract

**Background:**

Robot-assisted radical cystectomy (RARC) with intracorporeal diversion has been shown to be feasible in a few centers of excellence worldwide, with promising functional and oncologic outcomes. However, it remains unknown whether the complexity of the procedure allows its duplication in other non-pioneer centers. We attempt to address this issue by presenting our cumulative experience with RARC and intracorporeal neobladder formation.

**Methods:**

We retrospectively identified 62 RARCs in 50 men and 12 women (mean age 63.6 years) in two tertiary centers. Intracorporeal Studer neobladders were created, duplicating the steps of standard open surgery. Perioperative and postoperative variables and complications were analyzed using standardized tools. Functional and oncological results were assessed.

**Results:**

The mean operative time was 476.9 min (range, 310 to 690) and blood loss was 385 ml (200 to 800). The mean hospital stay was 16.7 (12 to 62) days with no open conversion. Perioperative complications were grade II in 15, grade III in 11, and grade IV in 5 patients. The mean nodal yield was 22.9 (8 to 46). Positive margins were found in in 6.4%. The 90- and 180-day mortality rates were 0% and 3.3%. The average follow-up was 37.3 months (3 to 52). Continence was achieved in 88% of patients. The cancer-specific survival rate and overall survival rate were 84% and 71%, respectively.

**Conclusions:**

A RARC with intracorporeal neobladder creation is safe and reproducible in ‘non-pioneer’ tertiary centers with robotic expertise with acceptable operative time and complications. Further standardization of RARC with intracorporeal diversion is a prerequisite for its widespread use.

## Background

Radical cystectomy with pelvic lymph node dissection is considered to be the most effective treatment of muscle-invasive bladder cancer. Moreover, it is an option in high-grade non-muscle-invasive disease refractory to intravesical instillation therapy. In the majority of cases, the most popular choice of diversion is either an ileal conduit or an orthotopic neobladder. Open radical cystectomy is still regarded as the gold standard treatment for muscle-invasive bladder cancer and high-risk non-muscle-invasive bladder cancer. However, minimally invasive radical cystectomy techniques have recently been gaining popularity and robot-assisted technique is used in most cases nowadays. Robot-assisted radical cystectomy (RARC) has been shown to be equivalent to open radical cystectomy in terms of oncological and functional outcomes and to be superior when it comes to perioperative outcomes, such as hospital stay and blood loss [1–5]. Surrogate parameters, such as nodal yield and positive surgical margins have been used to compare oncological outcomes with the open technique [2, 5]. Much criticism of RARC has been directed at urinary diversion, since most surgeons have performed it extracorporeally to shorten operating time. Hence, RARC and urinary diversion could at best be regarded as a hybrid approach. Jonsson and associates [6] have pioneered the technique of intracorporeal urinary diversion, creating both neobladders and ileal conduits completely intracorporeally. The technique is to be credited as an almost identical replication of open surgical principles. Recently, the group reported the oncologic and functional outcomes of their RARC cohort showing comparable results to open series [7]. Equally, Goh *et al.* presented remarkable outcomes using a modification of the aforementioned technique [8]. As such, RARC and intracorporeal urinary diversion are technically feasible with excellent outcomes. However, these observations are limited to very few pioneering centers worldwide. To date, it is unknown whether RARC and intracorporeal diversion are reproducible by other tertiary centers with robotic expertise. In particular, intracorporeal neobladder formation is regarded as technically challenging and potentially time-consuming with a steep learning curve [9, 10]. Herein, we present a homogenous series of patients who underwent RARC and subsequent intracorporeal neobladder formation, with the techniques described by Wiklund and colleagues [9, 10]. Herewith, we aim to demonstrate that a standardized technique of RARC and intracorporeal neobladder is safely reproducible and teachable, given adequate robotic experience.

## Methods

We retrospectively identified 62 patients who underwent RARC and orthotopic ileal neobladder formation in two academic centers, Eberhard-Karls University Tübingen, Germany, and Ankara Atatürk Training & Research Hospital, Turkey, for the period October 2009 to October 2014. A total of 300 radical cystectomies were performed during this period. One surgeon from each center who was experienced in open radical cystectomy as well as robotic prostatectomy performed the RARC with intracorporeal neobladder construction. Institutional review board approval was given before data analysis was started (# 081/2014R). Indications for RARC were muscle-invasive transitional carcinoma and non-muscle-invasive disease refractory to intravesical instillation treatment. Both men and women were included in the analysis. Patients receiving ileal conduit diversion were excluded. Other exclusion criteria were non-transitional-carcinoma histology, coagulopathy, pre-existing incontinence, cerebrovascular disease, and severe pulmonary dysfunction, rendering robotic surgery impossible. Patients with previous intra-abdominal surgeries or radiotherapy were not excluded. Data analyzed included perioperative variables (operating time, blood loss, hospital stay), standardized complication reporting using the Clavien-Dindo system [11], pathology reporting, and functional as well as oncologic outcomes. Furthermore, 90 and 180-day mortality are reported.

### Surgical technique

The RARC was performed using a three-arm Da Vinci S system (Intuitive Sunnyvale, CA, USA). One optical trocar was placed 2 cm above the umbilicus and a 0 degree telescope was used in all cases. The two remaining robotic trocars were placed a handbreadth lateral to the umbilicus and two more 12 mm assisting trocars were inserted 5 cm above the anterior-superior iliac spine. Finally, a 5 mm trocar was placed in the right upper abdomen. The RARC was then performed as described previously in a steep Trendelenburg position. Nerve sparing in both men and women was attempted whenever oncologically sound. Extended pelvic node dissection included the external and internal as well as the common iliac nodes, the obturator fossa, and the presacral area, as described previously. Para-aortic nodes were only removed when enlarged, in order to preserve the hypogastric plexus as much as possible. The bladder specimen and the lymph nodes were put into impermeable retrieval bags until removal at the end. Both ureters were clipped early and the left ureter was transposed below the sigmoid mesocolon. They were then tagged to the lateral abdominal wall until reimplantation. The robot was undocked and the operating table was flattened. The robot was redocked and a 50 cm loop of terminal ileum was isolated approximately 25 cm proximal to the ileocecal valve. The most dependent part was opened and anastomosed to the urethral stump using a running suture [6, 9]. The ileal loop was then discontinued using a 60 mm Endo-GIA stapler (Covidien, Mansfield, MA, USA). Bowel continuity was then restored using a stapled side-to-side anastomosis. The isolated loop was consequently opened according to Studer, leaving a 15 cm afferent segment. The posterior plate was then reconstructed using a 3/0 absorbable suture in a running fashion. The neobladder was then folded asymmetrically using a technique previously described by Wiklund [6, 9]. A 20 Fr Foley catheter was then advanced into the reservoir and its water-tightness was tested. The ureters were conjoined using the Wallace technique with a 4/0 absorbable suture. Single-J stents were placed over guide wire and the ends were advanced through the wall of the reservoir. Both ureters were then anastomosed to the afferent limb using a 4/0 absorbable suture. Stents were advanced to the skin through the 12 mm trocar and two drains were placed. Specimens were ultimately retrieved through a separate incision. (Specimens were delivered via the vagina in women.) Incisions were closed in layers.

### Statistical analysis

Data are descriptively reported using mean and standard deviation.

## Results

In total, 62 RARCs with intracorporeal neobladder formation were identified in 50 men and 12 women. The mean age was 63.6 years (41 to 80). The mean body mass index was 25.5 kg/m [2] (19 to 34). Studer neobladders were hand-sewn in all cases, replicating the open technique. Neoadjuvant chemotherapy was administered in nine patients with presumably advanced stage (Table 1). All procedures were completed without open conversion. The mean operating time was 476.9 min (310 to 690) and blood loss was 385 ml (200 to 800) while the mean diversion time was 183.8 minutes (140 to 300). The mean hospital stay was 16.7 (12 to 62) days. In the last ten patients, the mean diversion time was 146.6 min with an overall surgical time of 372.5 min (310 to 423). Bilateral nerve sparing was performed in all women and in 46 out of 50 men (92%). Perioperative complications were grade II in 15, grade III in 11, and grade IV in 5 patients. There were no complications associated with patient positioning (Table 2). The overall complication was 50%. Minor (Clavien I & II) and major (Clavien ≥ III) complication rates were 24.2% and 25.8% respectively. The postoperative tumor stage was carcinoma *in situ* in 4, pT0 in 6, pT1 in 5, pT2 in 23, pT3 in 18, pT4 in 6, and N+ in 15 patients and all were high-grade disease. The mean nodal yield was 22.9 (8 to 46). Positive margins were found in 6.4% of patients and all were positive ureteric margins. Concomitant prostate cancer was found in 14 men (28%). The Gleason score was 3 + 3 in nine, 3 + 4 in four, and 4 + 3 in one patient, respectively (Table 3). All but one man had a negative margin (positive margin rate 7.1%). However, to date there has not been any biochemical recurrence. The 90 and 180-day mortality rates were 0% and 3.3%. The average follow-up period was 37.3 months (3 to 52). Daytime continence could be achieved in 88% of patients while complete nighttime continence was noted in 58.1% of patients. Spontaneous erections were reported from 27 men (54%). Ureteric strictures developed in 8.3% of patients and were the most frequent long-term complication. Metastatic disease developed in 21% of patients and all of them received chemotherapy. Ten patients have died as a result of metastatic disease during the follow-up period, whereas five patients died from other non-cancer-related disease. The cancer-specific survival rate and overall survival rate were 84% and 71%, respectively (Table 4).

**Table 1:** **Patients’ characteristics**

Men (%)	50 (80.6)
Women (%)	12 (19.4)
Age, years, mean (range)	63.6 (41 to 80)
Body mass index, mean (range)	25.5 (19 to 34)
**Preoperative stage (%)**	
Carcinoma *in situ*	4 (6.5)
T1	3 (4.8)
T2	50 (80.6)
T3	4 (6.5)
T4	1 (1.6)
**Preoperative grade (%)**	
Low	0 (0)
High	62 (100)
Neoadjuvant chemotherapy (%)	9 (14.5%)
Previous Bacillus Calmette-Guérin instillation (%)	8 (12.9%)

**Table 2:** **Perioperative and postoperative outcome**

Operating time, min, mean (range)	476.9 (310 to 690)
Diversion time, min, mean (range)	183.8 (144 to 300)
Blood loss, ml, mean (range)	385 (200 to 800)
Conversion (%)	0 (0)
Hospital stay, days, mean (range)	16.73 (12 to 62)
**Nerve sparing, bilateral (%)**	
Men (%)	46/50 (92)
Women (%)	12/12 (100)
**Complications (Clavien) (%)**	
I	0 (0)
II	15 (24.2)
III	11 (17.7)
IV	5 (8.1)
V	0 (0)

**Table 3:** **Pathology characteristics**

**Postoperative pT-stage (%)**	
Carcinoma *in situ*	4 (6.5)
T0	6 (9.7)
T1	5 (8)
T2	23 (37.1)
T3	18 (29)
T4	6 (9.7)
**Postoperative grade (%)**	
Low	0 (0)
High	56 (100)
**pN stage (%)**	
N0	47 (75.8)
N+	15 (24.2)
Lymph node count (range)	22.9 (8 to 46)
Positive margins, %	6.4
**Concomitant prostate cancer (%)**	14 (22.6)
Gleason score (%)	
Gleason 6	9 (64.3)
Gleason 7a (Gleason 3 + 4)	4 (28.6)
Gleason 7b (Gleason 4 + 3)	1 (7.1)
Positive margins (prostate)	7.1%

**Table 4:** **Functional and oncologic outcomes**

Follow-up, months, mean (range)	37.3 (3 to 52)
**Continence, %**	
Daytime	88
Nighttime	55.1
Erectile dysfunction (%)	46
Recurrences (%)	1 (1.6)
Distant metastasis (%)	13 (21)
Cancer-specific survival (%)	84
Overall survival (%)	71

## Discussion

Robot-assisted radical cystectomy has been increasingly advocated as a viable alternative to open radical cystectomy. Despite a clear advantage with respect to perioperative morbidity and postoperative recovery, RARC is still not widely used. This is in sharp contrast to the widespread use of robotic radical prostatectomy. Initial criticism was focused on the lack of oncologic outcome data. Even though ultimate evidence may still be missing, recent reports confirm the long-term safety and efficacy of laparoscopic radical cystectomy as well as of RARC [5]. Surrogate parameters, such as nodal yield and margin status have been used to show equivalent oncological outcomes. Secondly, RARC has always been considered a very lengthy procedure with the potential risk of complications associated with the steep Trendelenburg position. This may be true for the initial series; however, contemporary data indicate competitive surgical times [9]. Thirdly, RARC and neobladder diversion has been deemed a cumbersome procedure, consisting of a robotic part and an open extracorporeal part for urinary diversion. Hence, many surgeons have questioned the point of performing RARC first followed by open diversion, as this removes most advantages, such as smaller wound incision and reduced bowel exposure. Wiklund and colleagues have pioneered the technique of intracorporeal neobladder formation following RARC. They follow the principles of open surgery in creating a Studer neobladder, including proper folding [12]. Their approach has evolved, leading to technical modifications and a reduction of complications and operation times. In a cumulative analysis of 70 patients with a median follow-up of 30.3 months, they found negative margins in 98.6% of cases. Relevant complications occurred in 31.4% of patients at 30 days and 18.6% of patients at >30 days. At 90 days, the overall complication rate was 58.5%. The recurrence-free, cancer-specific, and overall survival rates at 24 months were 80.7%, 88.9%, and 88.9%, respectively. Daytime continence and satisfactory sexual function or potency at 12 months ranged between 70% and 90% in both men and women. These results are well in line with contemporary open-surgery results [7]. A retrospective comparison with open diversion in the framework of the International Robotic Cystectomy Consortium, including 935 patients, further confirmed the safety of intracorporeal diversion by showing a lower risk of postoperative complication, including gastrointestinal complications in these patients [13]. Notably, only 61 patients had undergone intracorporeal neobladder formation in this comparative study. In the USA, similar approaches have been described. Although a shorter follow-up time was recorded with fewer patients, excellent results have been reported [8]. Herein, we present our cumulative experience with RARC and intracorporeal neobladder formation performed in two tertiary referral centers with robotic expertise. Surgery has been performed according to previously published principles. As such, our series represents a real-life experience of intracorporeal diversion outside of the pioneering centers. Many findings by the pioneer group have been corroborated and their results have been widely replicated. Hence, RARC and intracorporeal neobladder formation may be safely implemented in a clinical routine whenever adequate robotic experience is available. To the best of our knowledge, our report describes the largest series of RARC and intracorporeal diversion apart from the Karolinska group. The fact that the results are comparable to those of the inventors of the approach may be of particular value when it comes to assessment of teaching and overcoming the learning curve. Importantly, the approach has now been transformed into a standardized robotic procedure with well-defined steps. Obviously, our surgical times in the initial cases were long, owing to the learning curve. However, over time, the average operative times could be reduced to less than 6 hours. This is in line with the demonstration by various studies that robotic cystectomy and intracorporeal neobladder construction can be achieved safely with a structured approach in a high volume center [9, 14].

The functional outcomes of patients who underwent robotic cystectomy is quantified by continence and sexual function. In our cases, continence is defined as usage of less than one pad a day. The continence outcome is usually measured at 12 months after surgery, as the assessment is more accurate after the neobladder has achieved its functional capacity. Similarly, sexual function is also measured at 12 months.

From our experience, another important element to a successful RARC with intracorporeal neobladder that provides patients with good oncological, functional and perioperative outcomes is a dedicated robotic team. An RARC with intracorporeal diversion is extremely complex and can be intimidating for any young robotic surgeon. It helps to have a dedicated robotic team, as familiarization is important not just for the surgeon, but also for the bedside assistants and scrub team, as well as the theater staff. The bedside assistant should be familiar with standardized surgical steps; the scrub team should be well equipped with all the instruments, sutures, and staplers required, and the operating theater staff should be prepared in terms of proper patient positioning and necessary protection. In an operation of such high complexity, teamwork from all the members of the team involved is crucial, to ensure smooth sailing surgery and to minimize complications and conversion rate, and shorten the operating time.

With adequate experience, the refinement of surgical techniques also contributes to the safety and feasibility of an intracorporeal neobladder. A hallmark of a good ileal neobladder is large capacity for storage, low pressure, and high compliance for continence, and the ability to voluntarily void with minimal residual urine [15]. A conventional spherical pouch has been proven urodynamically to be of high volume and low pressure and gives excellent functional outcomes [16]. Various different types of ileal neobladder have been used in patients who underwent open radical cystectomy and neobladder reconstruction. One study showed similar perioperative and functional outcomes using a non-spherical pouch [17]. Some of these less complicated technique can be adapted into robotic intracorporeal neobladder construction and could further shorten the operating time without compromising outcomes.

In our experience, exposure to complex intracorporeal surgery of a lesser degree such as laparoscopic or robotic intracorporeal ileal ureter is helpful in familiarization with the steps involved in intracorporeal bowel resection and anastomosis [18].

Our results have shown the safety and reproducibility of this complex technique with comparable operating time, complications, functional, and oncologic outcomes to those of the pioneer centers. However, we face similar limitations to those experienced by previous authors , such as retrospective nature, limited number of patients, and follow-up durations. However, current results seem to favor robotic cystectomy with intracorporeal urinary diversion; a conclusion which can only be established eventually with a randomized study of adequate power.

## Conclusions

With the advancement of technology and refinement of surgical technique, it is a matter of time before more surgical teams will embark on robotic cystectomy and intracorporeal neobladder construction. A standardized technique with a dedicated robotic team in a high volume center makes robotic intracorporeal neobladder construction a feasible and reproducible technique.

### Consent

Written informed consent was obtained from the patients for the publication for this report and any accompaying imaging.

## Abbreviations

RARC: robot-assisted radical cystectomy.
